# Evaluation of gray matter perfusion in episodic migraine using voxel-wise comparison of 3D pseudo-continuous arterial spin labeling

**DOI:** 10.1186/s10194-018-0866-y

**Published:** 2018-05-23

**Authors:** Zhiye Chen, Xiaoyan Chen, Mengyu Liu, Mengqi Liu, Lin Ma, Shengyuan Yu

**Affiliations:** 1 Department of Radiology, Chinese PLA General Hospital, 28 Fuxing Road, Beijing, 100853 China; 2 Department of Radiology, Hainan Branch of Chinese PLA General Hospital, Beijing, 100853 China; 3 Department of Neurology, Chinese PLA General Hospital, 28 Fuxing Road, Beijing, 100853 China

**Keywords:** Brain, Episodic migraine, Gray matter, Magnetic resonance imaging, 3D pseudo-continuous arterial spin labeling

## Abstract

**Background:**

Although previous studies have demonstrated that structural and functional abnormalities in episodic migraine (EM), less is known about altered brain perfusion in the EM. The aim of this study is to investigate altered gray matter perfusion in EM using a 3D volumetric perfusion imaging.

**Methods:**

Fifteen EM patients and 15 normal controls (NC) underwent structural and 3D pseudo-continuous arterial spin labeling (3D pc-ASL). The structural images were segmented using DARTEL methods and the generated normalized T1 tissue probability maps were used to coregister the cerebral blood flow (CBF) images, which would further be performed with standardization using Fisher Z Transformation. Voxel-wise analysis was applied to CBF map with Z standardization, and the Z value of the abnormal brain region was extracted and performed with correlation with the clinical variables.

**Results:**

The increased CBF value located in the left Brodmann 38 (BA38) and no significantly decreased CBF value were detected in EM. HAMD scores presented significantly positive correlation with the CBF value of the left BA38.

**Conclusion:**

The current study indicated that the pattern of cerebral hyperperfusion may elucidate the neurogenic mechanism in the EM genesis, and 3D pc-ASL technique would non-invasively provide valuable cerebral perfusion information for the further pathophysiological and neuropsychological study in EM.

## Background

Migraine is a common primary headache disorder, which was the second largest contributors of disability-adjusted life-years in the Global Burden of Disease Study [1]. Migraine episodes are characterized classically by unilateral, throbbing headache, frequently associated with nausea, vomiting, photophobia, phonophobia, or allodynia [2]. The pathogenesis of migraine is not completely understood. Cortical spreading depression (CSD) has been considered to account for migraine aura and to be a migraine trigger via trigeminal sensory afferents activation [3]. Cerebral or meningeal vasodilation and potential perivascular release of vasoactive substances may lead to headache generation in migraine [4]. Advanced imaging studies have provided more insights into migraine pathophysiology and migraine-related dysfunctions. The brainstem has been considered to play a pivotal role in the first phase of a migraine attack. Limbic pathways and cognitive processing network have participated in migraine processing. Hyperexcitability of visual cortex and thalamus in migraine may be associated with photophobia and allodynia respectively. Interictal abnormalities such as altered grey volume, white matter lesions, altered neural activity and functional connectivity in migraine and their correlation with migraine duration/frequency suggested migraine may be a progressive brain disorder [5, 6]. However, whether the altered structure and function is the cause or the result of migraine attack is still a debate.

Previous perfusion imaging studies using xenon 133 intra-arterial injection method, single photon emission computed tomography (SPECT), CT perfusion, and perfusion-weighted MR imaging (PWI) have been conducted for migraine research. The hypoperfusion during aura in hemisphere contralateral to neural deficit did not fulfill vascular distribution, supporting a neurogenic rather than vascular explanation for migraine aura [7–10]. However, The perfusion status of interictal migraine were inconsistent [11–13]. The previous perfusion studies were generally case reports or small sample-sized, perhaps limited by the radioactivity or contrast of the scanning.

3D pseudo-continuous ASL (3D pc-ASL) was a novel non-enhancement perfusion sequence on MR750 3.0 T(GE Healthcare, Milwaukee, WI, USA). Advantage of this technique included 3D acquisition, spiral k-space filling, FSE pulse sequence and non-invasive labelling technique without MRI contrast injection, which would further expand the clinical application range of ASL. ASL has been adopted for migraine aura and hypoperfusion was detected, consistent with former perfusion studies [14, 15]. However, so far, very few studies used ASL for interictal migraine research and the results differed [16–18].

The aim of this study is detect the pattern of altered cerebral perfusion at interictal stage of episodic migraine (EM) without aura. We prospectively obtained high resolution structural images and 3D pc-ASL images from 15 EM patients and 15 normal controls(NC). Voxel-wise comparison of CBF maps were performed between EM and NC, and the correlation analysis were applied between the CBF values of the abnormal brain regions and the clinical variables.

## Methods

### Subjects

Fifteen EM patients and 15 normal controls (NC) were recruited from the International Headache Center, Department of Neurology, Chinese PLA General Hospital. The inclusion criteria should be fulfilled as follows: 1) EM is defined as migraine attack days being less than 15 days per month. The definition of migraine refers to 1.1 Migraine without aura in ICHD 3beta [2]; 2) no migraine preventive medication used in the past 3 months; 3) absence of other subtypes of headache, chronic pain other than headache, severe anxiety or depression preceding the onset of headache, psychiatric diseases, etc.; 4) absence of alcohol, nicotine, or other substance abuse; and 5) patient’s willingness to engage in the study. NCs were recruited from the hospital’s staff and their relatives. Inclusion criteria were similar to those of patients, except for the first items. NCs should never have had any primary headache disorders or other types of headache in the past year. The exclusion criteria were the following: cranium trauma, illness interfering with central nervous system function, psychotic disorder, and regular use of a psychoactive or hormone medication. General demographic and headache information were entered in our headache database. All the patients were given with the Visual Analogue Scale (VAS) and the migraine disability assessment scale (MIDAS), and all the subjects received anxiety and depression evaluation by using the Hamilton Anxiety Scale (HAMA) [19], the Hamilton Depression Scale (HAMD) [20], respectively.

The study protocols were approved by the Ethical Committee of Chinese PLA General Hospital and complied with the Declaration of Helsinki. Written informed consent was obtained from all participants according to the approval of the ethics committee of the local institutional review board. MRI scans were taken in the interictal stage at least three days after a migraine attack for EM patients. All the subjects were right-handed and underwent conventional MRI examination to exclude the subjects with cerebral infarction, malacia, or occupying lesions. Alcohol, nicotine, caffeine, and other substances were avoided for at least 12 h before MRI examination.

### MRI acquisition

Images were acquired on a GE 3.0 T MR system (DISCOVERY MR750, GE Healthcare, Milwaukee, WI, USA) and a conventional eight-channel quadrature head coil was used. All subjects were instructed to lie in a supine position, and formed padding was used to limit head movement. The structural images were acquired by a three-dimensional T1-weighted fast spoiled gradient recalled echo (3D T1-FSPGR) sequence generating 360 contiguous axial slices [TR (repetition time) = 7.0 ms, TE (echo time) = 3.0 ms, flip angle = 15^。^, FOV (field of view) = 25.6 cm × 25.6 cm, Matrix = 256 × 256, NEX (number of acquisition) = 1]. Volumetric perfusion imaging was obtained using a pseudo-continuous ASL tagging scheme with a 3D interleaved spiral FSE readout (3D spiral FSE ASL) with parameters as: TR/TE = 5128/15.9 ms, flip angle = 111^o^, FOV = 20 cm × 20 cm, x, y matrix = 1024 × 8 (spiral acquisition), slice thickness = 3.0 mm. The labeling duration was 1.5 s, and post-labeling delay time (PLD) was 1.5 s.Oblique axial T2-weighted imaging (T2WI), T1 fluid-attenuated inversion recovery (T1-FLAIR) and diffusion weighted imaging (DWI) were also acquired. All imaging protocols were identical for all subjects. No obvious structural damage and T2-visible lesion were observed on the conventional MR images.

### MR image processing

#### Generating CBF maps

3D pc-ASL data, including perfusion weighted images and proton density-weighted images, was processed using Functional tools (version:9.4.05) on GE Advanced Workstation 4.5. Fifty axial CBF images were acquired based on the following equation according to the reported lieratures [21–26]:

$$ f\kern0.5em =\kern0.5em \frac{\lambda }{2\alpha {T}_{1b}\left(1-{e}^{\frac{\tau }{T_{1b}}}\right)}\frac{\left({s}_{con}\kern0.5em -\kern0.5em {S}_{lbe}\right)\left(1-{e}^{\frac{t_{sat}}{T_{1g}}}\right)}{S_{ref}}e\frac{w}{T_{1b}} $$

*f*, flow; λ = 0.9 (brain–blood partition coefficient); α = 0.85 (labeling efficiency); *T*_*1b*_ = 1.6 s (the T1 value ofblood); *T*_*1g*_ = 1.2 s (the T1 value of gray matter); τ = 1.5 s (labeling duration); *S*_*con*_, *S*_*lbe*_ and *S*_*ref*_, the singnal of control, label and reference images, respectively; *t*_*sat*_ = 2 s (the saturation time for proton density images); w, post-labeling delay.

### 3D structural segmentation and CBF maps normalization

**Fig. 1:**
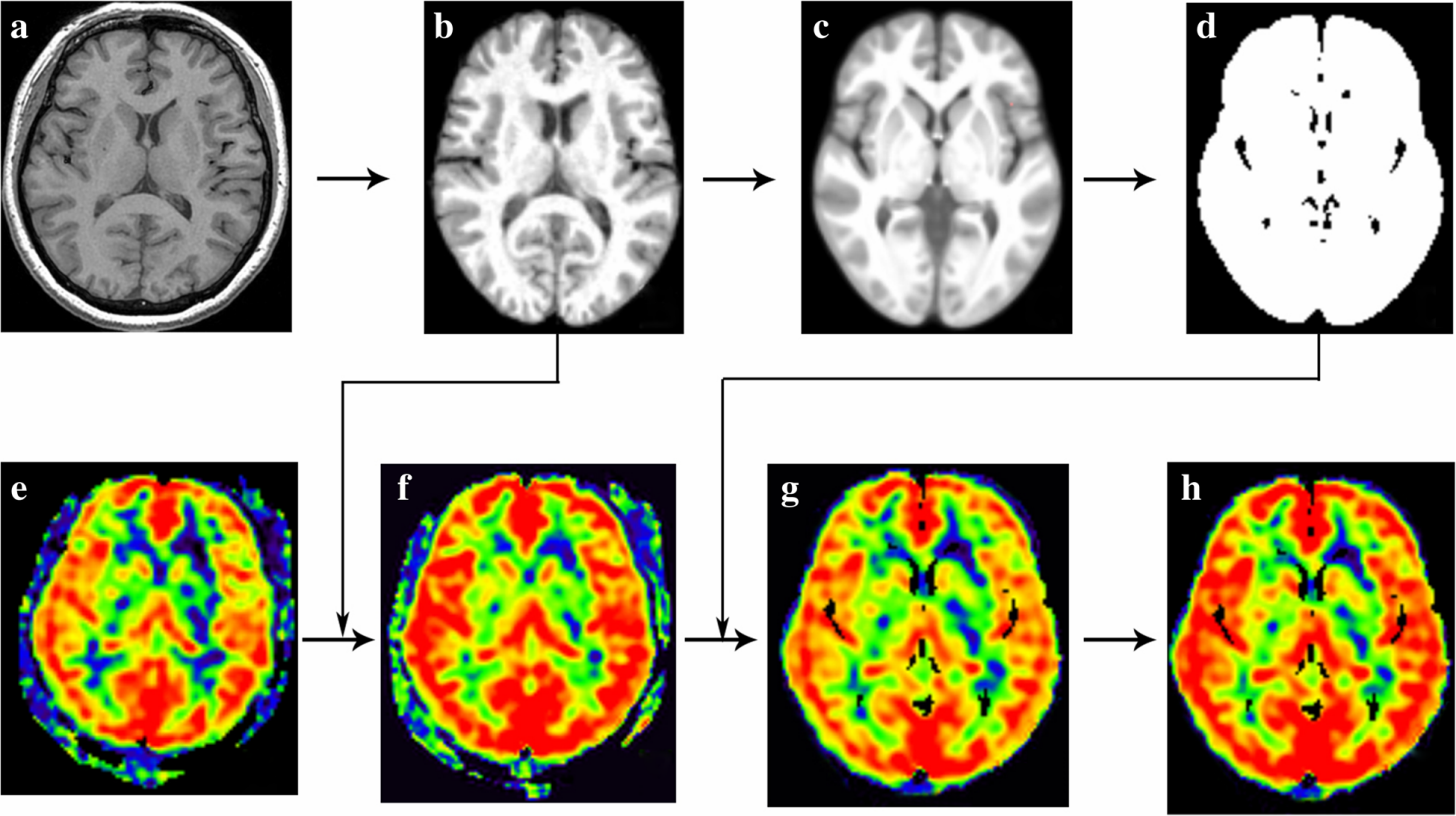
Methods of CBF normalization and standardization. The individual raw T1 images (**a**) were segmented and generated T1 tissue probability maps (**b**), which would be used to generate average T1 tissue probability maps (**c**) and brain mask (**d**). The individual CBF maps (**e**) was coregistered by the T1 tissue probability maps, which would generate normalized CBF maps (**f**). The brain mask was applied with the normalized CBF maps in order to extract the brain tissue (**g**), and the Z transformation was performed with the normalized CBF maps to standardize the CBF maps (**h**)

All MR image data were processed using Statistical Parametric Mapping 12 (SPM 12) (http://www.fil.ion.ucl.ac.uk/spm/) running under MATLAB 7.6 (The Mathworks, Natick, MA, USA) to perform structural segment [27] and CBF maps normalization [28, 29]. The image processing included following steps: (1) The structural images were segmented into grayand white matter tissue probability maps (GM-TPM and WM-TPM) using DARTEL methods, which simultaneously generated the normalized T1 tissue probabilitymaps (T1-TPM); (2) All the T1-TPM wereused to generate average T1-TPM and further generate brain mask; (3) The individual CBF maps were spatially normalize into strandard Montreal Neurologic Institute (MNI) stereotaxic space by coregistering with the individual T1-TPM and resampled into 1.5 × 1.5 × 1.5 mm^3^ isotropic size, which would generated individual normalized CBF maps (nCBF); (4) The individual nCBF maps were warped by the brain mask to extract brain tissue; (5) The individual normalized CBF maps with brain extraction (bet_nCBF)were performed Z transformation to avoid individual hemodynamic variation, and spatially smoothed with a 6-mm isotropic Gaussian kernel (Fig. 1).

**Fig. 2:**
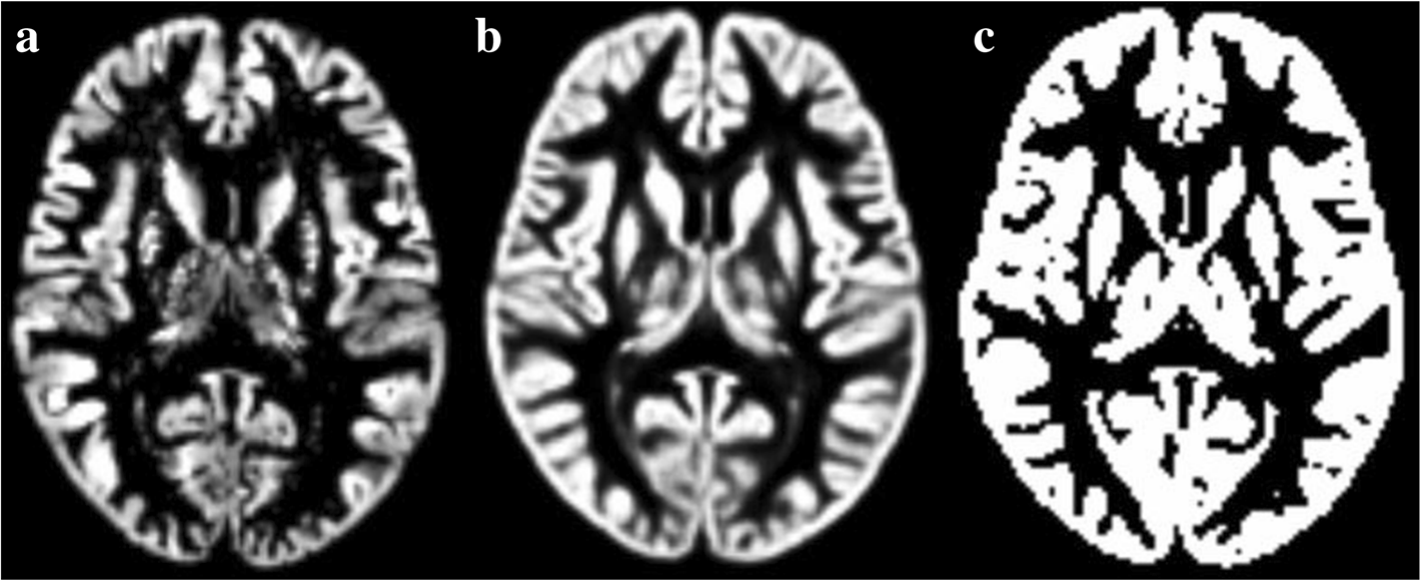
The generation of customized gray matter mask. **a**, individual gray matter tissue probability maps; **b**, customized gray matter template; **c**, customized gray matter mask

All the individual GM-TPM (including EM and NC) were used to generate customized GM template using DARTEL tool software package. The customized GM template was transformed as GM mask (Fig. 2), which was used as an explicit mask in the voxel-based analysis for 3D pc-ASL data.

### Statistical analysis

The statistical analysis was performed by using PASW Statistics 18.0. The data with non-normal distribution presented by median (minimum, maximum) and the data with normal distribution presented as mean ± standard deviation. The quantitative data (including age, HAMA, HAMD) was performed with independent samples T test, and the qualitative data (including sex) was applied with Chi-Square test. The Pearson correlation was performed with the data with normal distribution, and the Spearman correlation was performed with the data with non-normal distribution. Significant difference was set at a *P* value of < 0.05.

Voxel-wise comparison of volumetric perfusion was performed by the SPM 12 software. The factorial design was set as Two-sample t-test with age and sex as covariates. The customized gray matter mask was selected as the explicit mask, and all the voxels in the customized gray matter mask were performed with voxel-wise comparison. The minimal number of contiguous voxels was based on the expected cluster size.

## Results

### Demography and neuropsychological test

The current study included 15 EM patients (F/M = 11/4) and 15 NC (F/M = 11/4). The age (EM, 32 ± 10.62 years old; NC, 38 ± 9.56 years old) and sex presented no significant difference between EM and NC. The headache variables of EM were listed as follows: the disease duration 10(0.5,21) years, headache frequence 3(1,10) per month, the mean interictal time 10.27 ± 4.73 days after last migraine attack at scanning, VAS 8(6,10), sleep quality 1(0,3) and MIDAS 11.53 ± 12.44. The HAMA and HAMD score significantly increased in EM patients (16.13 ± 10.51 and 15.73 ± 2.91, respectively) compared with that in NC (9.73 ± 3.39 and 11.4 ± 7.52, respectively) (*P* = 0.001 and 0.000, respectively).

### Comparison of gray matter perfusion between EM and NC

**Fig. 3:**
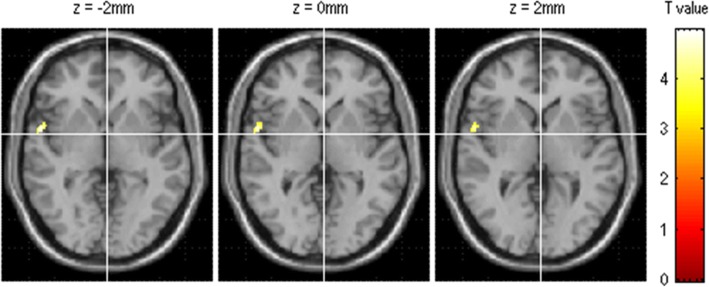
The brain region with hyperperfusion located in the left superior temporal (BA 38)in EM patients compared with NC

The brain region with increased perfusion located in the left Brodmann 38 (BA38) (the left superior temporal) (MNI coordinate, 57 5–2; *P*_*uncorr*_ value, 0.000; cluster size, 143) (Fig. 3). There was no significant decreased perfusion in EM compared with NC.

### Correlation analysis between CBF value of positive brain regions and clinical variables

HAMD scores were positively related with the CBF value of the left BA38 (*r* = 0.529, *P* value = 0.043). The other clinical variables, including VAS scores, disease duration, MIDAS score, headache frequency, sleep quality and HAMA scores, showed no significant correlation with the CBF value of the left BA38 (Table 1).

**Table 1:** Correlation analysis between the CBF value of the brain region with hyperperfusion and the clinical variables

	Left BA38		
	r value	*P* value	
VAS^a^	− 0.479	0.071	
DD^a^	−0.333	0.225	
MIDAS^b^	−0.082	0.772	
HF^a^	0.093	0.742	
SQ^a^	0.462	0.083	
HAMA^b^	−0.286	0.302	
HAMD^b^	0.529	0.043	
MoCA^a^	0.422	0.117	

*VAS* visual analogue scale, *DD* disease duration (years), *MIDAS* migraine disability assessment scale, *HF* headache frequency (per month), *SQ* sleep quality, *HAMA* Hamilton Anxiety Scale, *HAMD*, Hamilton Depression Scale, *MoCA* Montreal Cognitive Assessment.^a^, Spearman correlation analysis, ^b^, Pearson correlation analysis

## Discussion

This study adopted 3D pc-ASL, a novel non-enhancement perfusion sequence to detect the pattern of altered cerebral perfusion in interictal phase of EM in a China headache center. By this method, we found increased perfusion in the left BA38 (the left superior temporal) in EM compared with NC and a positive correlation of HAMD scores with the CBF value of the left BA38.

Perfusion imaging studies have been conducted for migraine research for decades. For migraine with aura (MA), hypoperfusion during aura in hemisphere with posterior predominance contralateral to neural deficit was usually detected by using xenon 133 intra-arterial injection method [10],single photon emission computed tomography (SPECT) [7], CT perfusion [9], perfusion-weighted MR imaging (PWI) [8], and ASL [8, 14, 15] followed by hyperperfusion at headache phase [7, 10, 14]. For prolonged hemiplegic migraine, hemispheric hyperperfusion [30] and normal perfusion [31] have also been observed using PWI method. The perfusion status during headache attack of migraine without aura (MO) varied more [32, 33], which may largely depend on when the imaging is done and the course of the migraine attack.

The interictal perfusion studies varied too. One study using 133Xe inhalation method showed that a derangement of the cerebral perfusion was present in both MA and MO, suggesting they were due to the same disease process [12] while another study only found abnormal mean hemispheric blood flows or disturbed intra-hemispheric rCBF patterns in MA rather than MO [13].Later studies using SPECT found decreased CBF in patients at interictal phase of MA, which often corresponded to the site of headache and the topography of transient neurological symptoms [34, 35]. In a recent study using PWI, interictal hyperperfusion was observed in the inferior and middle temporal gyrus in MO patients, hypoperfusion was seen in the postcentral gyrus and in the inferior temporal gyrus in MA patients and in the inferior frontal gyrus in MO patients [11].A research group using pc-ASL found increased rCBF within the primary somatosensory cortex (S1) in adult migraineurs as well as in pediatric and young adult migraineurs [17]. However, other two ASL studies did not find differences in resting CBF between MA, MO and NC [16, 18].

Most of previous perfusion studies were case reports and few were small cohorts. The diversity of disease severity and accompanied disorders, the scanning methods and timing of examination may lead to the inconsistency of perfusion studies at headache and interictal phase. Compared to the earlier perfusion techniques for migraine research using xenon 133 intra-arterial injection, SPECT, CT perfusion, PET, newer techniques using PWI and ASL have advantages in superior spatial resolution, increased sensitivity, and being nonradioactive. ASL has advantages over PWI in that no contrast is needed [36, 37] and has high spatial resolution. Therefore, ASL may be an ideal technique for migraine research since repeated ASL can be acquired during each phase of migraine attack without harm to patients.

Areas with altered interictal perfusion may reflect local interictal differences in neuronal metabolism or activity, or the presence of some degree of interictal cerebrovascular dysregulation in migraineurs [11].Like some previous perfusion studies, this study observed regional CBF alteration in interictal phase of MO which did not fulfill vascular distribution, supporting that migraine was a primary neurogenic disorder. In this study, we found only one brain region with hyperperfusion: left BA38(the left superior temporal gyrus) in EM patients.

The left BA38 is located in temporal pole(TP), which is supposed to be a convergence zone integrating information from auditory, somato-sensorimotor, visual, olfactory, language, paralimbic structures and default-semantic network, suggesting its participation in autonomic regulation, multisensory, memory, and emotional processing [38]. Neuroimaging studies have provided much evidence supporting an important role of TP in migraine pathophysiology. Gray matter density within left TP significantly decreased interictally and increased ictally for MO compared with NC [39], implicating a key role in cyclical recurrence of migraine attacks. The TP was hyperexcitable with painful heat stimulation and showed increased resting-state connectivity with hypothalamic in interictal migraine patients, indicating that TP may be involved in the interictal hypersensitivity to pain, smell and light [40, 41].In another fMRI study, increased average regional homogeneity values in TP showed significantly positive correlations with disease duration of MO [42]. During odour stimulation in a H(2)(15)O-PET study, migraineurs showed significantly higher activation than controls in the left TP, suggesting a role in olfactory hypersensitivity of migraine [43].A recent study found that enhancing excitability of the TP with non-invasive anodal transcranial direct current stimulation normalized abnormal interictal visual information processing in migraineurs [44], supporting TP as a possible therapeutic target to relieve hypersensitivity of migraine. Our study further provides evidence of TP perfusion abnormality in interictal migraine and this structure needs deeper investigation. TP and superior temporal gyrus have been shown to be involved in major depressive disorders [45, 46]. In our study, the hyperperfusion of left BA38 and its positive correlation with HAMD may be associated with the genesis of multisensory hypersensitivity and mood disorder of migraine.

The interictal perfusion alteration in this study supported migraine as a central nervous dysfunction and may provide biomarkers for migraine diagnosis and treatment. The mechanism of how the perfusion in this region changed is still unknown and needs to be further investigated. In addition to the alteration of the left BA38 as reported by this study, previous interictal studies usually found volume and functional alteration in brain stem, thalamus, anterior cingulate cortex, insula, prefrontal cortex, etc., indicating the involvement of multiple brain regions in migraine processing network [5, 6]. However, we did not find perfusion changes in those regions. Aa a matter of fact, the perfusion may not necessarily change in consistent with grey volume or neural function [47].

This study has some limitations. Firstly, we only included MO patients in interictal phase, thus we could not speculate whether there’s difference of the interictal brain between MO and MA patients. Secondly, we only scanned once for a patient and the dynamic perfusion changes during different phase of a migraine attack and post-attack were not presented. In the future, we may repeatedly scan migraine patients at more timing points. Thirdly, the sample size was not large enough, thus we did not analyze the influence to positive brain areas by some clinical parameters such as headache laterality and headache-free time. Lastly, masking of the CBF maps with the segmented T1 image did not sufficiently address the issue of partial volume correction (PVC) in ASL data because of the sensitive to noise and errors in the partial volume estimates, and the further advanced PVC methods should be performed to balance the spatial and smooth effect in the PV estimates [48].

## Conclusion

In conclusion, this study revealed that the interictal hyperperfusion in left temporal pole and vlPFC may reflect the neural metabolism abnormality regarding multi-dimensional pain processing in migraine. The pattern of cerebral hyperperfusion at interictal migraine may elucidate the neurogenic mechanism in the EM genesis. 3D pc-ASL technique would non-invasively provide valuable cerebral perfusion information for the further pathophysiological and neuropsychological study in EM.

## Abbreviations

3D pc-ASL: 3D pseudo-continuous arterial spin labeling
BA38: Brodmann 38
bet_nCBF: normalized CBF maps with brain extraction
CBF: cerebral blood flow
EM: episodic migraine
GM-TPM: gray matter tissue probability map
HAMA: Hamilton Anxiety Scale
HAMD: Hamilton Depression Scale
MA: migraine with aura
MIDAS: migraine disability assessment scale
MO: migraine without aura
NC: normal controls
nCBF: normalized CBF maps
PWI: perfusion-weighted imaging
SPECT: single photon emission computed tomography
T1-TPM: T1 tissue probability maps
TP: temporal pole
VAS: visual analogue scale
WM-TPM: white matter tissue probability map
